# High-Resolution Image Analysis Reveals a Decrease in Lens Thickness and Cone Density in a Cohort of Young Myopic Patients

**DOI:** 10.3389/fmed.2021.796778

**Published:** 2021-12-16

**Authors:** Xiaoyu Xin, Qingge Guo, Shuai Ming, Changgeng Liu, Zhongfeng Wang, Bo Lei

**Affiliations:** ^1^Department of Ophthalmology, Henan University People's Hospital, Henan Provincial People's Hospital, Zhengzhou, China; ^2^Henan Eye Institute, Henan Eye Hospital, Henan Provincial People's Hospital, Zhengzhou, China; ^3^State Key Laboratory of Medical Neurobiology and MOE Frontiers Center for Brain Science, Institutes of Brain Science, Fudan University, Shanghai, China; ^4^Department of Ophthalmology, Zhengzhou University People's Hospital, Zhengzhou University, Zhengzhou, China

**Keywords:** myopia, retina, cone, lens, adaptive optics

## Abstract

**Purpose:** To study the association between axial length (AL) and the thickness of the lens, retina, choroid, and cone density with swept-source optical coherence tomography (SS-OCT) and an adaptive optics (AO) fundus camera.

**Design:** A prospective cross-sectional study.

**Methods:** This study included 136 eyes in 68 subjects. SS-OCT was used to quantify the thickness of the lens, ganglion cell complex (GCC) layer, inner nuclear layer (INL), outer retinal layer (ORL), and choroid layer. Adaptive optics was used to quantify spatial features of the cone photoreceptors, including density, spacing, regularity, and dispersion. The associations among the AL and the thickness of lens, retina, choroid, and cone features were evaluated with linear regression.

**Results:** With the severity of myopia, the increased AL was associated with thinning of the lens (*P* < 0.001, 95% CI: −100.42 to −49.76). The thickness of the ORL and choroid decreased significantly (all *P* < 0.001), whereas the thickness of the GCC and INL decreased only in the outer ring (both *P* < 0.01). There was a significant correlation between the cone density/spacing and AL (both *P* < 0.001). Although cone density was reduced from 25,160/mm^2^ to 19,134/mm^2^ in the inner region and from 17,458/mm^2^ to 13,896/mm^2^ in the outer region, the best-corrected visual acuity (BCVA) was 20/20 or greater.

**Conclusions:** We found that the lens thickness (LT), ORL, and cone density decreased in myopia. While decreasing cone density and ORL thickness should be related to axial elongation, decreasing of LT might imply intrinsic physical accommodation. These results provide further morphological changes of myopia.

## Introduction

Myopia is a common refractive condition where parallel rays entering the eye are imprinted in front of the retina rather than focusing on it when ocular accommodation is relaxed (1). The prevalence of myopia is increasing dramatically on a global basis (2), particularly in East Asia, where the prevalence of myopia reaches >80% in young adults (3). It is assumed that 50% and 10% of the world population will develop myopia and high myopia (HM), respectively (4). Myopia is regarded as a major global public health problem (3). Accordingly, understanding the anatomical changes of myopia is of great importance for understanding the mechanisms of this condition, as well as making strategic plans to control it.

Recently, it has been generally believed that myopia may be the result of a disruption of the balance between axial elongation and loss of refraction power of the cornea and lens (5, 6). It was reported that corneal power became stabilized early in life, at approximately 1–2 years after birth (7, 8). Consequently, the importance of lens power and axial length (AL) in myopia is exaggerated (9). To maintain an emmetropization status, AL continues to elongate while the lens tends to be thin and flatten during human growth and development (3). However, when the lens reaches a limit where it can no longer compensate for the growth of the axis, myopia occurs (7, 9).

With the development of myopia, the fundus also changes. Several studies reported that choroidal thickness (10, 11) and choroidal blood perfusion (10, 12) reduced dramatically in HM eyes. Meanwhile, reductions in retinal thickness and vascular density have been demonstrated (13, 14). These changes observed in HM eyes were regarded mainly as the consequence of axial elongation (15, 16). The primary function of the choroid is to supply oxygen and nourishment to the outer retina (17). When the choroid thickness and blood perfusion decrease, the outer retina is affected, which may lead to dysfunction of the photoreceptors (18).

However, reports concerning whether the thickness of the lens and whether the photoreceptors are changed concurrently during the progression of myopia are limited, partially because of lacking applicable technology that could reveal the detailed morphological changes. To explore the changes of cone photoreceptors in myopia, we used an adaptive optics (AO) fundus camera to measure the parameters of the cone photoreceptors in patients. Simultaneously, the thicknesses of the lens, retina, and choroid were obtained from high resolution swept-source optical coherence tomography (SS-OCT), which presented higher penetration up to 6 mm and made as detailed a measurement as possible. We analyzed the relationship between AL and the lens thickness (LT), choroidal thickness, retinal thickness, and spatial features of the cone photoreceptors in a cohort of young myopic patients.

## Methods

### Subjects

A total of 68 subjects between the ages of 21 and 32 years were recruited from Henan Provincial People's Hospital from December 2020 to February 2021. Approval for the study was obtained from the Ethics Committee of Henan Eye Hospital. All participants signed written informed consent after being informed about the nature and possible consequences of the project, in compliance with the tenets of the Declaration of Helsinki.

All the participants underwent the collection of disease history and a comprehensive ophthalmic examination, including best-corrected visual acuity (BCVA), refraction error (non-cycloplegic), non-contact tonometer (NT-530, NIDEK, Gamagori, Japan) for intraocular pressure (IOP), slit-lamp, and fundus photography (DRS, Padova, Italy). Axial length was measured (IOL Master 500; Carl Zeiss Meditec AG, Jean, Germany). The spherical equivalent (SE) of all eyes was calculated followed by the spherical power plus half of the negative cylinder power. The inclusion criteria were 20–35 years of age; BCVA 20/20 or better; 10–21 mmHg of IOP; no eye tremor, and good fixation. The exclusion criteria were the use of any eye drops recently, abnormal physical development in eyes, any history of general diseases, ocular diseases and eye surgeries, and pregnant and lactating women.

### Swept-Source Optical Coherence Tomography and Swept-Source Optical Coherence Tomography Angiography Measurements

All subjects were imaged using SS-OCT (VG200D SVision Imaging, Henan, China) with a central wavelength of 1,050 nm and a scanning speed of 200,000 A-scans per second. The system provided axial resolutions of 5 μm and transverse resolutions of 20 μm. Only high-quality scans which had signal strength >8 (0 = poor, 10 = good) were selected. The data of both eyes of the subjects were collected. All scans were performed by the same experienced operator.

**Figure 1:**
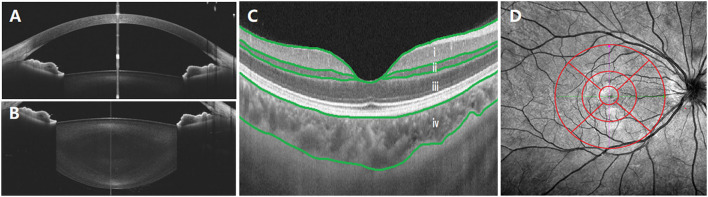
Examination of central corneal thickness (CCT), anterior chamber depth (ACD), lens thickness (LT), choroid, and retina. **(A)** CCT and ACD; **(B)** LT; **(C)** optical coherence tomography (OCT) image. The built-in software in swept-source OCT (SS-OCT) automatically divided fundus structure into four layers: (i) ganglion cell complex (GCC) layer; (ii) inner nuclear layer (INL); (iii) outer retinal layer (ORL); (iv) choroid layer. **(D)** OCT angiography (OCTA) image. The white vertical line is “corneal reflex” in **(A,B)**, the red region is the Early Treatment Diabetic Retinopathy Study (EDTRS) grid in **(C)**. CCT, central corneal thickness; ACD, anterior chamber depth; LT, lens thickness; OCT, optical coherence tomography; SS-OCT, swept-source optical coherence tomography; OCTA, optical coherence tomography angiography; EDTRS, Early Treatment Diabetic Retinopathy Study.

In anterior segment scanning, two sets of images (the anterior chamber was included in the cornea group) were obtained from 18 consecutive radial B-scans (Star 18 Line R32 mode) of the cornea/lens by the axial shifting of the focal planes on the cornea and the lens, respectively. During imaging, the examiner observed the position of the eyes to achieve optimal alignment, which was defined as a “corneal reflex” (an optically vertical line through the center of the image, Figures 1A,B) was seen. The central corneal thickness (CCT), anterior chamber depth (ACD), and LT were measured with built-in software. The images were excluded from the analysis when their quality from one eye was poor.

Posterior segment scanning mode (Angio 6 × 6 mm, 512 × 512 R4 modes) was used to obtain macular SS-OCTA (a 6 × 6 mm radial scan centered on the fovea). In this mode, a total of 512 lines were scanned each time. In total, 512 A-scans were performed for each line, and each line was scanned four times. Retinal/choroidal thickness was automatically divided into four layers (Figure 1C) with the built-in software: (i) ganglion cell complex (GCC) layer, perpendicularly from the inner edge of the retinal nerve fiber layer to the outer edge of the inner plexiform layer; (ii) inner nuclear layer (INL), perpendicularly from the inner edge of the INL to the outer edge of the INL; (iii) outer retinal layer (ORL), perpendicularly from the inner edge of the outer plexiform layer to the inner edge of the RPE; (iv) choroid layer, perpendicularly from the outer edge of the RPE to the choroid–sclera junction. A retinal/choroidal thickness map was established based on the Early Treatment Diabetic Retinopathy Study (ETDRS) grid (Figure 1D), which was centered on the fovea, separating the region into nine subfields by three concentric circles: an inner circle (diameter of 1 mm), a middle circle (diameter of 3 mm), and an outer circle (diameter of 6 mm). The inner ring (between the inner and middle circles) and the outer ring (between the middle and outer circle) were divided into four quadrants: superior, temporal, inferior, and nasal. The built-in SS-OCT software automatically calculated the average values of the eight regions, including four quadrants from the inner and outer rings. The images of two eyes were poor and were excluded from the analysis.

### AO Measurements

**Figure 2:**
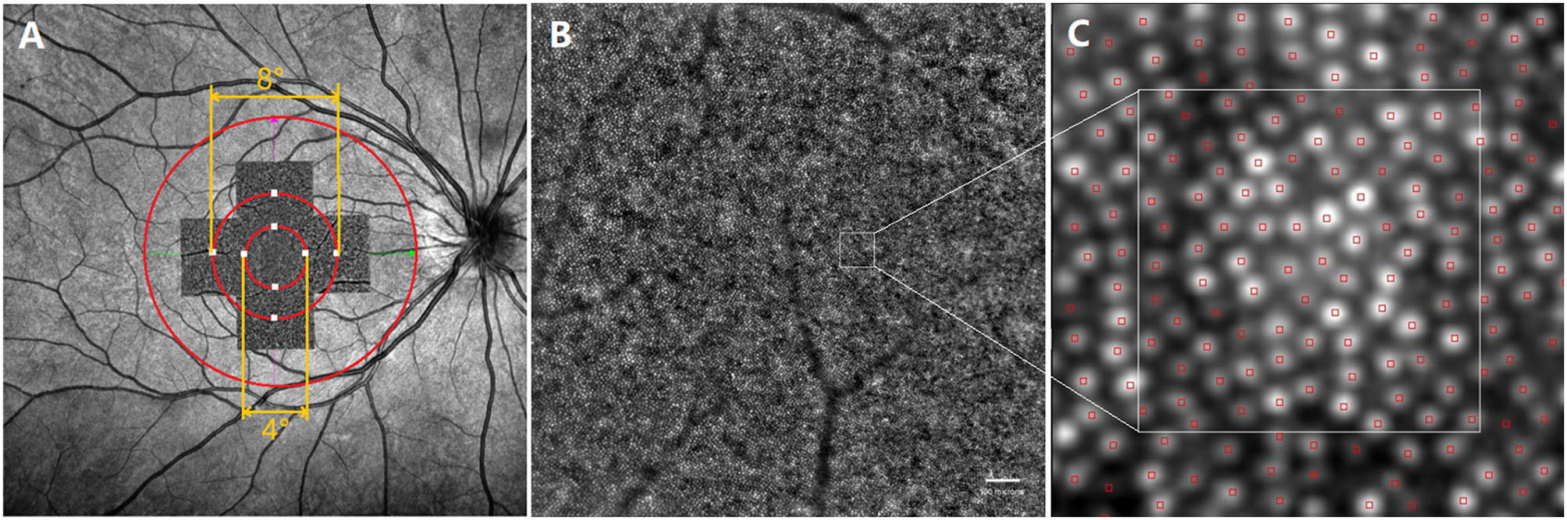
Representative images of swept -optical coherence tomography angiography (SS-OCTA) and adaptive optics (AO). **(A)** The AO image is superimposed on the fundus image by SS-OCTA. The AO montage with a window size of 10° × 10° was created centered on the fovea. The eight white squares shown in the figure represent the measured locations, including four for the inner region (the circle with a diameter of 4°) and four for the outer region (the circle with a diameter of 8°); **(B)** one of the images that made up the AO montage. ROI, white square, represented acquisition window (the size of 100 × 100 μm); **(C)** The white square is a magnified view of an ROI. SS-OCTA, swept-optical coherence tomography angiography; AO, adaptive optics; ROI, region of interest.

An AO fundus camera (rtx1, Imagine Eyes, Orsay, France) was used to reveal the spatial features of cone photoreceptors. One captured field of imaging was 4° × 4° which was equivalent to 1,200 × 1,200 μm. The initial coordinate was (0°, 0°), which corresponded to the fovea. The subjects were asked to consecutively fixate at 3° of eccentricity along the four meridians of superior, temporal, inferior, and nasal, to image the parafoveal regions. After the image acquisition, five pictures were analyzed with the built-in software (i2k Retina Pro) to generate a montage size of approximately 10° × 10° which was equivalent to 3 × 3 mm. The inner ring of the ETDRS was the area between the inner circle (diameter 1 mm) and the middle circle (diameter 3 mm), corresponding to the view of the AO montage image (3 × 3 mm) (Figure 2, red cycle).

The spatial features of the cone photoreceptors were analyzed with software provided by the manufacturer (AO Detect 0.2; Imaging Eyes, Orsay, France). The cones were analyzed at 2° (inner region) and 4° (outer region) eccentricity along the four meridians of superior, temporal, inferior, and nasal. Three 100 × 100 μm regions of interest (ROIs) avoiding blood vessels were chosen. The spatial characteristics of cones, including density, spacing, regularity, and dispersion were recorded. Cone density was defined as the number of cells per square millimeter, cone spacing was defined as the center-to-center spacing of adjacent cones, cone regularity was defined as the percentage of cells that had five to seven neighbor cells, and cone dispersion was defined as the spread of cones, which was the coefficient of variation of the cone spacing. The average values of the three parameters were obtained. The AO images of 40 eyes were poor and were excluded from the analysis.

### Statistical Analysis

Statistical analyses were performed using SPSS (version 21.0; IBM SPSS, Chicago, Illinois, USA). Age differences were analyzed by Spearman's correlation. Pearson's correlation and regression were used to analyze the relationship between AL and the biological parameters of anterior and posterior segments. A *P*-value of 0.05 was chosen to denote statistical significance.

## Results

### General Information

A total of 136 eyes were included. The demographics are presented in Table 1, and the ocular biometric parameters are presented in Supplementary Table 1. There were 52 eyes from 26 men and 84 eyes from 42 women including 37 emmetropia and low myopia (EM/LM, +0.50 to −2.75 D of the sphere), 52 moderate myopia (MM, −3. 00 to −5.75 D of the sphere), and 47 HM (HM, ≤ −6.00 D of the sphere) eyes. The mean age of the patients was 24.07 ± 2.33 years, with a range between 21 and 32 years.

**Table 1:** Patient demographics.

**Variable**	**Total (*n* = 136)**
Age, years	
Range	21–32
Mean ± SD	24.07 ± 2.33
Min, Median, Maximum	21, 24, 32
Gender	
Men	52 (38%)
Women	84 (62%)
SE, D	
EM/LM	37 (27%)
Range	+0.50 to −2.75
Mean ± SD	−1.60 ± 1.12
Min, Median, Maximum	−2.75, −2.00, 0.00
MM	52 (38%)
Range	−3. 00 to −5.75
Mean ± SD	−4.92 ± 0.81
Min, Median, Maximum	−5.75, −5.00, −3.00
HM	47 (35%)
Range	≤ -6.00
Mean ± SD	−7.69 ± 1.20
Min, Median, Maximum	−11.50, −7.25, −6.00

*Data values are n (%) or mean ± SD. SD, standard deviation; SE, spherical equivalent; EM/LM, emmetropia and low myopia; MM, moderate myopia; HM, high myopia*.

### Associations Between Axial Length and Ocular Parameters

**Figure 3:**
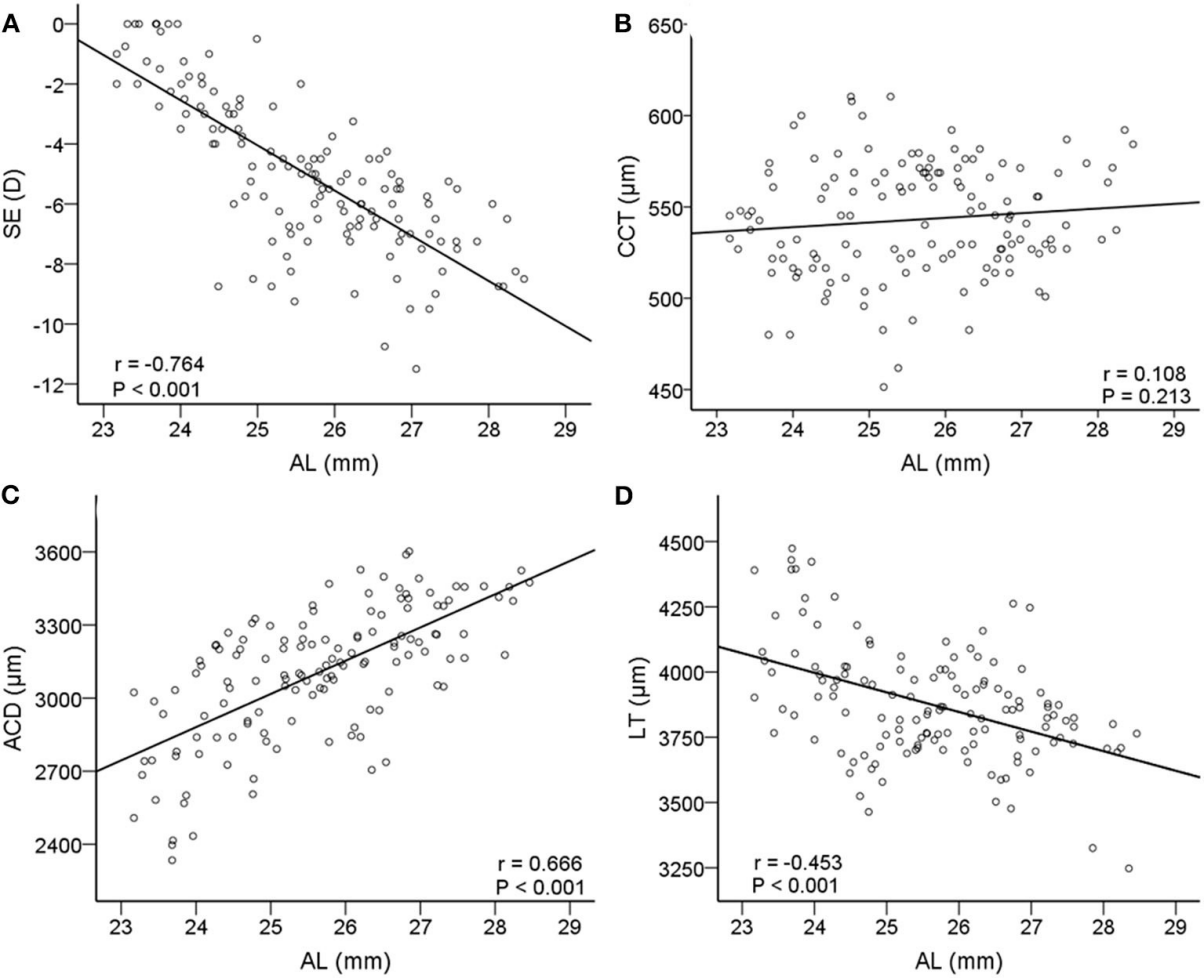
Correlations between axial length (AL) and other relative biometric parameters. Scatterplots showing AL vs. **(A)** spherical equivalent (SE); **(B)** CCT; **(C)** ACD; **(D)** LT. AL, axial length; SE, spherical equivalent; CCT, central corneal thickness; ACD, anterior chamber depth; LT, lens thickness.

We investigated linear relationships between AL and SE. Axial length was negatively correlated with SE (*r* = −0.764, *P* < 0.001, 95% CI: −1.72 to −1.29, Figure 3). In Figure 3, with the increase of AL, ACD increased significantly (*r* = 0.666, *P* < 0.001, 95% CI: 100.20 to 162.61) and LT decreased significantly (*r* = −0.453, *P* < 0.001, 95% CI: −100.42 to −49.76), while CCT did not change (*r* = 0.108, *P* = 0.213, 95% CI: −1.48 to 6.58).

**Figure 4:**
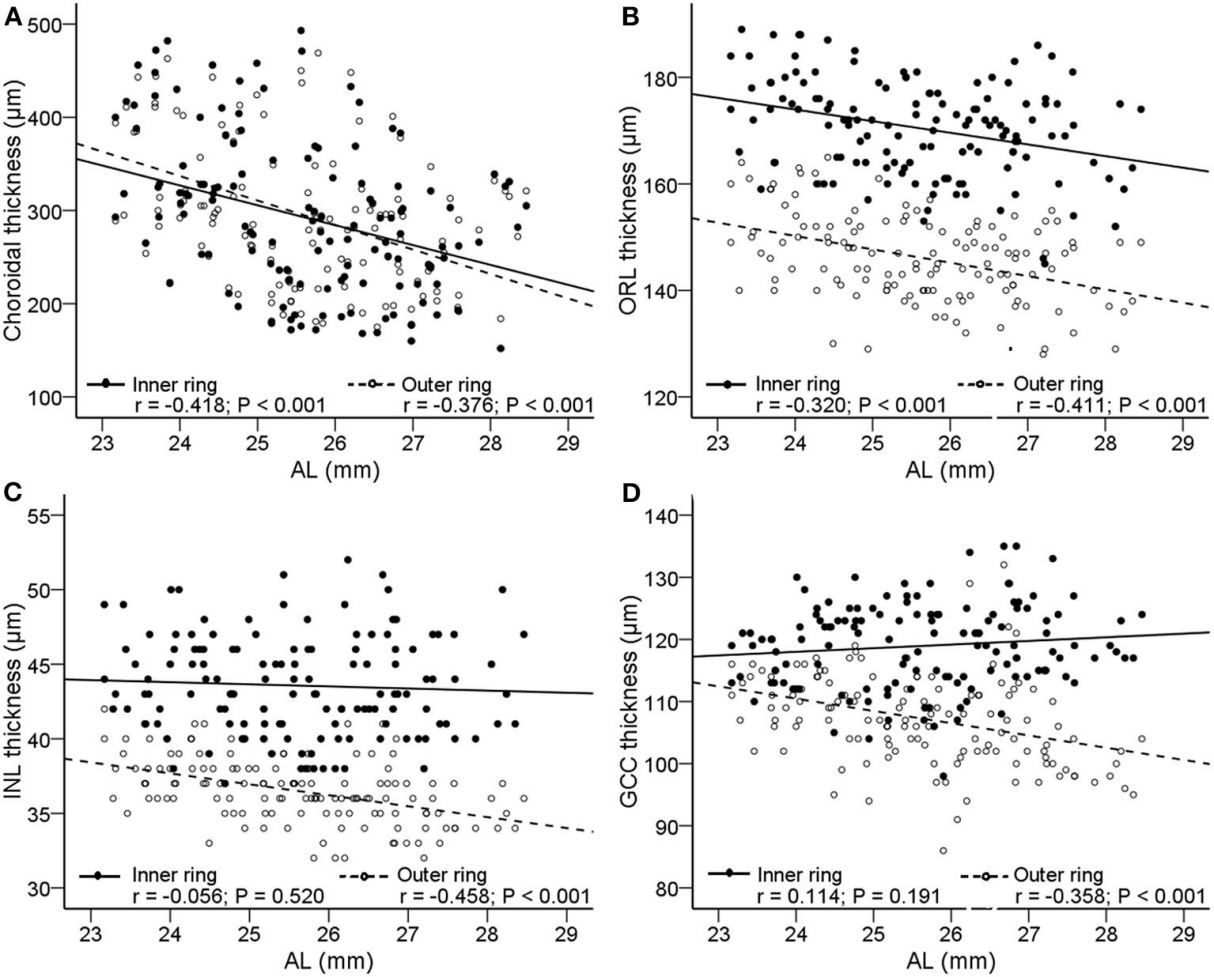
Correlations between AL and the thickness of choroid and retina. Scatterplots showing AL vs. **(A)** choroidal thickness; **(B)** outer retinal layer (ORL); **(C)** inner nuclear layer (INL); **(D)** ganglion cell complex (GCC). The *solid lines* and *filled circles* represent the results in the inner ring. The *dashed lines* and *open circles* represent the results in the outer ring. AL, axial length; ORL, outer retinal layer; INL, inner nuclear layer; GCC, ganglion cell complex.

Figure 4 shows the scatter plots and linear regression of retinal and choroidal thickness as functions of AL. With axial elongation, the thickness of ORL and choroid decreased significantly both in the inner ring and outer ring (*r* = −0.418 to −0.320, all *P* < 0.001, Figure 4), whereas the GCC and INL layers tended to decrease in the outer ring (*r* = −0.458 to −0.358, both *P* < −0.01, Figure 4). In addition, we found that as AL increased, the GCC thickness of the superior, temporal, and inferior sectors in the outer ring decreased, but that of the nasal sector had no change (Supplementary Table 2).

### Associations Between Axial Length and the Spatial Features of Cones

**Figure 5:**
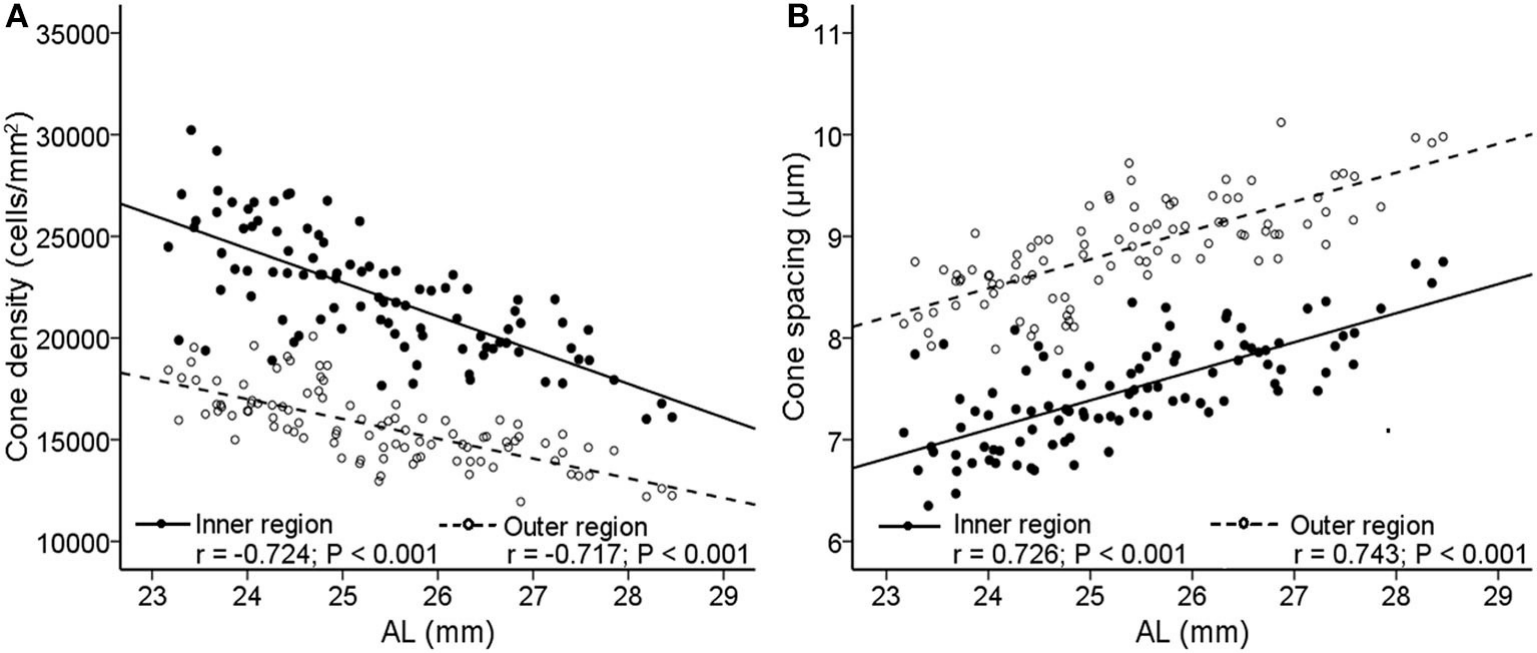
Correlations between AL and cone photoreceptor spatial distribution features. Scatterplots show AL vs. **(A)** cone density; **(B)** cone spacing. The *solid lines* and *filled circles* represent the results in the inner region. The *dashed lines* and *open circles* represent the results in the outer region. AL, axial length.

The cone spatial distribution has four characteristics: density, spacing, regularity, and dispersion. Axial length elongation was significantly correlated with density and spacing both in the inner and outer regions (*r* = −0.724 to.743, all *P* < 0.001, Figure 5; Table 2), while was not related to the regularity and dispersion (both *P* > 0.05, Table 2).

**Table 2:** Correlations between AL and cone spatial distribution features.

	**Inner region**		**Outer region**	
	***r***	***P***	***r***	***P***
Density	−0.724	**<0.001**	−0.717	**<0.001**
Spacing	0.726	**<0.001**	0.743	**<0.001**
Regularity	−0.168	0.101	−0.139	0.176
Dispersion	0.372	0.053	0.194	0.057

*Significant difference bolded*.

### The Relationship Between the Cone Spatial Features and the Thickness of Choroid and Retina

We further analyzed the association between the spatial characteristics of cone photoreceptors with the thickness of the choroid and retina. We found that thickness of the choroid/ORL was significantly correlated with cone density (*r* = 0.297 to.332, both *P* < 0.01, Table 3) and cone spacing (*r* = −0.311 to −0.292, both *P* < 0.01, Table 3), but not with cone regularity and dispersion (all *P* > 0.05, Table 3). However, with the decrease of INL, cones were less regular (*r* = 0.224, *P* = 0.028, 95% CI: 0.01 to 0.18, Table 3) and more dispersed (*r* = −0.228, *P* = 0.025, 95% CI: −0.15 to −0.01, Table 3), whereas GCC was not associated with cone density and spacing (both *P* > 0.05, Table 3).

**Table 3:** Correlations between cone features and the thickness of choroid and retina.

	**Cone density, cells/mm** ^**2**^		**Cone spacing**, **μm**		**Cone regularity, %**		**Cone dispersion, %**	
	***r***	***p***	***r***	***p***	***r***	***p***	***r***	***p***
Choroidal thickness, μm	0.297	**0.003**	−0.292	**0.004**	0.052	0.617	0.016	0.880
ORL thickness, μm	0.332	**0.001**	−0.311	**0.002**	0.195	0.057	−0.221	**0.031**
INL thickness, μm	0.111	0.282	−0.101	0.327	0.224	**0.028**	−0.228	**0.025**
GCC thickness, μm	0.051	0.621	−0.046	0.655	0.157	0.127	−0.160	0.119

*Significant difference bolded*.

## Discussion

We evaluated the changes of major biometric parameters of the myopic eyes by SS-OCT and cone photoreceptor spatial features by AO. This study was a comprehensive evaluation of the changes of the anterior and posterior segment parameters simultaneously in myopic eyes. We found that among the changes of the refractive system and fundus structure in young myopic adults, the changes with regards to the lens and cones were particularly prominent, in addition to the choroidal thickness.

Our findings are in agreement with previous studies that AL was negatively correlated with LT (19, 20), positively correlated with ACD (20), and not correlated with CCT (21, 22). Central corneal thickness, ACD, and LT are important components of AL. Before axial myopia occurs, the image could still focus on the retina through adaptive thinning of the lens. However, when the thinning of the lens fails to neutralize the growing axis of the eye (9), the image is focused in front of the retina, which may be the so-called irreversible true axial myopia. Because an accommodative lens may play a key role in the development of myopia, it is crucial to understand the changes of the lens during this process. While such data are still limited, partially because of lacking adequate and accurate detecting instruments.

During human growth and development, the thickness and shape of the lens change by producing new protein fibers (7, 20, 23, 24). Studies have shown that most children are far-sighted in the first few years of their life. With the growth of the eye, the AL gradually increases and the lens becomes relatively thinner to achieve a state of emmetropia (23, 24). There were a few reports concerning lens changes in myopia, nevertheless, the conclusions were controversial. Some studies showed that thinning of the lens was correlated with myopia (20, 24, 25), while some suggested that there was no correlation between LT and myopia (26, 27). Our findings are consistent with the former. A possible explanation for the difference in the results may be the methods or the subjects enrolled in these studies, such as the age of the patients and instruments used. In our study, the mean age of the participants was 24.07 ± 2.33 years, which was younger than other studies (26, 27). On the other hand, we used SS-OCT, which provided micron-scale accuracy, and we strongly believe it was better than instruments applied in previous studies. In most studies, myopia was thought to be caused mainly by the lengthening of the vitreous cavity (28), while little attention was paid to the changes of the lens. Our study provided solid evidence that reducing LT may be crucial in counteracting axial elongation functionally. During the progress of myopia, thinning of LT may, to some extent, compensate for the elongation of AL, which helps the eye maintain better vision. However, thinning of the lens should have certain limitations. When the limitation was reached, a focused image would be formed in front of the retina and near sight happens. Therefore, the importance of the changes of LT in myopia, especially in the earlier stages, was exaggerated.

Concerning the posterior segment, several studies have shown the relationship between the thinning of the retina and choroid with increasing myopia (29–32). Our findings further confirmed that of previous studies that thinning of ORL and choroid occurs in the inner and outer rings. In addition, we found that there was a significant negative correlation between AL and GCC or INL in the outer ring, but there was no correlation in the inner ring. The findings supported the opinion proposed by Lim et al. and Liu et al. that the thinning of the retina is more common in the outer ring (16, 33). In addition, as the AL increased, the GCC thickness of the superior, temporal, and inferior quadrants in the outer ring decreased, but the nasal quadrant did not change. A possible explanation was that as the eye stretched axially, the retina moved temporally away from the optic disc, which was also a reason for the formation of the tilted optic disc and the temporal crescent in high myopia.

We found that increased AL was significantly associated with decreased cone density and increased cone spacing in the inner and outer regions. One possible explanation was that the changes in cone photoreceptor distribution in young myopia were due to mechanical tension caused by axial elongation. The area covered by the retina was expanded along with the increase of the AL, which in turn caused the covering of a larger area with the same number of photoreceptors. Although cone density was reduced from 25,160/mm^2^ to 19,134/mm^2^ in the inner region and from 17,458/mm^2^ to 13,896/mm^2^ in the outer region in this cohort of patients, their BCVA, however, was 20/20 or greater. These data suggested that the decrease of cone density measured at 2 and 4 degrees of eccentricity did not affect the visual acuity in this cohort, even though the cone density at the fovea was still unknown. The notion was supported by our previous study where the cone system function was not affected in a similar cohort of myopia cases as tested by an electroretinogram (34). On the other hand, we did not see the association between AL and cone regularity, as observed in previous studies in myopia (18). It should be noticed that the current AO cannot ensure a high-quality image in each individual, even in young subjects. Factors that affect image acquisition include vitreous floc opacity, which is not uncommon in myopia patients. On the other hand, the quality of AO imaging is limited by pupil size and refractive compensation. Therefore, useful images were not obtained from all subjects. In addition, an AO retinal camera cannot resolve the cones in the center of the fovea because of the density, hence the software probably limits the closest analysis to two-degree eccentricity. Clearly, further research is necessary when an adequate standard is established.

In summary, we observed changes in the anterior and posterior segments in a cohort of myopic patients with high-resolution image systems. In addition to the decreasing of retinal and choroidal thickness, we found that the LT and cone density were remarkably decreased. Our study indicated that the change of the lens happened concurrently with the mosaic of cone photoreceptors. Thus, clinical assessment in myopia should consider these changes in the development of the common disorder, which may, in turn, result in further revealing their underlying mechanisms and developing novel interventions for this complicated condition.

## Data Availability Statement

The original contributions presented in the study are included in the article/Supplementary Material, further inquiries can be directed to the corresponding author/s.

## Ethics Statement

The studies involving human participants were reviewed and approved by the Ethics Committee of Henan Eye Hospital. The patients/participants provided their written informed consent to participate in this study. Written informed consent was obtained from the individual(s) for the publication of any potentially identifiable images or data included in this article.

## Author Contributions

BL conceived and designed this study. XX and QG collected the clinical samples and clinical data. XX and SM analyzed the sequencing data. XX collected the information and drafted and revised the manuscript. BL and ZW directed the work and finalized the manuscript. All authors contributed to the article and approved the submitted version.

## Funding

This work was supported by National Natural Science Foundation of China Grants (81770949 and 82071008) and the Henan Key Laboratory of Ophthalmology and Vision Science.

## Conflict of Interest

The authors declare that the research was conducted in the absence of any commercial or financial relationships that could be construed as a potential conflict of interest.

## Publisher's Note

All claims expressed in this article are solely those of the authors and do not necessarily represent those of their affiliated organizations, or those of the publisher, the editors and the reviewers. Any product that may be evaluated in this article, or claim that may be made by its manufacturer, is not guaranteed or endorsed by the publisher.
