# Supplementary material for: High-Resolution Image Analysis Reveals a Decrease in Lens Thickness and Cone Density in a Cohort of Young Myopic Patients
Source: Front Med (Lausanne). 2021 Dec 16;8:796778. doi: 10.3389/fmed.2021.796778 (PMC8716878; doi:10.3389/fmed.2021.796778)
Supplement: Supplementary file 1 [file Data_Sheet_1.docx]

| **SUPPLEMENTARY** **TABLE S1 \|** Measurements of the biometric parameters in myopia | | | |
| --- | --- | --- | --- |
| Parameters | Mean ± SD | Minimum | Maximum |
| AL, mm | 25.63 ± 1.32 | 23.17 | 28.46 |
| CCT, µm | 543.07 ± 31.26 | 451.3 | 610.4 |
| ACD, µm | 3102.57 ± 270.75 | 2334.7 | 3602.3 |
| LT, µm | 3874.92 ± 219.03 | 3247.5 | 4473.4 |
| GCC thickness, µm (inner ring) | 118.95 ± 6.82 | 98 | 135 |
| GCC thickness, µm (outer ring) | 107.26 ± 7.31 | 86 | 132 |
| INL thickness, µm (inner ring) | 43.57 ± 3.35 | 37 | 52 |
| INL thickness, µm (outer ring) | 36.49 ± 2.13 | 32 | 42 |
| ORL thickness, µm (inner ring) | 170.42 ± 9.06 | 145 | 189 |
| ORL thickness, µm (outer ring) | 146.15 ± 8.10 | 128 | 165 |
| Choroidal thickness, µm (inner ring) | 294.37 ± 83.36 | 152 | 493 |
| Choroidal thickness, µm (outer ring) | 292.33 ± 75.22 | 175 | 469 |
| Cone density, cells/mm^2^ (inner region) | 22146.81 ± 3013.21 | 16014 | 30226 |
| Cone density, cells/mm^2^ (outer region) | 15677.01 ± 1781.44 | 11951 | 20085 |
| Cone spacing, µm (inner region) | 7.49 ± 0.52 | 6.35 | 8.75 |
| Cone spacing, µm (outer region) | 8.87 ± 0.50 | 7.88 | 10.12 |
| Cone regularity, % (inner region) | 93.86 ± 1.53 | 89.50 | 97.17 |
| Cone regularity, % (outer region) | 94.59 ± 1.57 | 90.40 | 97.91 |
| Cone dispersion, % (inner region) | 11.68 ± 1.06 | 9.26 | 14.43 |
| Cone dispersion, % (outer region) | 10.89 ± 1.54 | 7.86 | 15.66 |

SD = standard deviation; AL = axial length; CCT = central corneal thickness; ACD = anterior chamber depth; LT = lens thickness; GCC = ganglion cell complex; INL = inner nuclear layer; ORL = outer retinal layer.

| **SUPPLEMENTARY** **TABLE S2A \|** Correlations between AL and choroidal/retinal thickness in the inner ring | | | | | | | | |
| --- | --- | --- | --- | --- | --- | --- | --- | --- |
|  | S | | T | | I | | N | |
|  | r | *P* | r | *P* | r | *P* | r | *P* |
| Choroid | -0.389 | **< 0.001** | -0.401 | **< 0.001** | -0.411 | **< 0.001** | -0.395 | **< 0.001** |
| ORL | -0.276 | **0.001** | -0.224 | **0.009** | -0.411 | **< 0.001** | -0.311 | **< 0.001** |
| INL | -0.092 | 0.288 | -0.048 | 0.586 | -0.023 | 0.790 | -0.015 | 0.865 |
| GCC | -0.039 | 0.654 | -0.127 | 0.145 | -0.047 | 0.592 | 0.190 | **0.028** |

Significant difference bolded.

S = superior; T = temporal; I = inferior; N = nasal; AL = axial length; ORL = outer retinal layer; INL = inner nuclear layer; GCC = ganglion cell complex.

| **SUPPLEMENTARY** **TABLE S2B \|** Correlations between AL and choroidal/retinal thickness in the outer ring | | | | | | | | |
| --- | --- | --- | --- | --- | --- | --- | --- | --- |
|  | S | | T | | I | | N | |
|  | r | *P* | r | *P* | r | *P* | r | *P* |
| Choroid | -0.303 | **< 0.001** | -0.378 | **< 0.001** | -0.386 | **< 0.001** | -0.362 | **< 0.001** |
| ORL | -0.330 | **< 0.001** | -0.248 | **0.004** | -0.440 | **< 0.001** | -0.478 | **< 0.001** |
| INL | -0.489 | **< 0.001** | -0.314 | **< 0.001** | -0.493 | **< 0.001** | -0.344 | **< 0.001** |
| GCC | -0.388 | **< 0.001** | -0.544 | **< 0.001** | -0.382 | **< 0.001** | -0.057 | 0.516 |

Significant difference bolded.

S = superior; T = temporal; I = inferior; N = nasal; AL = axial length; ORL = outer retinal layer; INL = inner nuclear layer; GCC = ganglion cell complex.
